# Preparing Children for Invasive Medical Cancer Treatment with “My Logbook”: Preliminary Results of a Pilot Study

**DOI:** 10.1007/s13187-024-02481-2

**Published:** 2024-08-07

**Authors:** Liesa J. Weiler-Wichtl, Verena Fohn-Erhold, Verena Rosenmayr, Rita Hansl, Maximilian Hopfgartner, Jonathan Fries, Carina Schneider, Kristina Herzog, Tobias Schellenberg, Barbara Schönthaler, Nicole Stember, Iris Lein-Köhler, Rahel Hoffmann, Alina Kollmann, Nicole Salzmann, Stefanie Essl, Katharina Pal-Handl, Verena Wasinger-Brandweiner, Sarah Rinner, Lisa Schubert, Sandra Lange, Ulrike Leiss

**Affiliations:** 1 Department of Paediatrics and Adolescent Medicine, General Hospital, Medical University of Vienna, Währinger Gürtel 18-20, 1090 Vienna, Austria; 2 Department of Developmental and Educational Psychology, Faculty of Psychology, University of Vienna, Vienna, Austria; 3 Childhood Cancer International – Europe (CCI-E), Vienna, Austria; 4 Elternhilfe für krebskranke Kinder e.V. Leipzig, Leipzig, Germany; 5 Department of Paediatric Oncology/Haematology, Charité-Universitätsmedizin Berlin, Berlin, Germany; 6 Central Hospital Bolzano, Bolzano, Autonomous Province of Bolzano Italy; 7 West German Proton Therapy Center Essen (WPE), Essen, Germany; 8 Department of Paediatric Haematology and Oncology, Saarland University Medical Center, Homburg, Germany; 9 Department of Psychosomatic Medicine and Psychotherapy, Medical Faculty, University of Leipzig, Leipzig, Germany; 10 Kepler Universitätsklinikum Linz Med Campus IV, Linz, Austria; 11 Paediatric Haematology and Oncology, University Children’s Hospital Münster, Münster, Germany; 12 Department of Childhood and Adolescent Medicine, University Clinic Salzburg, Salzburg, Austria; 13 Department of Childhood and Adolescent Medicine, University Hospital Vienna, Vienna, Austria; 14 St. Anna Children’s Hospital, Vienna, Austria; 15 Children’s Hospital, University of Würzburg, Würzburg, Germany; 16 Department of Pediatric Hematology and Oncology, University Hospital Frankfurt, Frankfurt, Germany; 17 Department of Clinical Psychology and Behavioral Neuroscience, Faculty of Psychology, Technical University Dresden, Dresden, Germany; 18 KOKON - Psychosocial and Mental Health in Pediatrics Lab, Rohrbach-Berg, Austria

**Keywords:** Cancer education, Pediatric oncology, Quality of care, Implementation research, Feasibility, Evidence-based interventions, Psychosocial care

## Abstract

Pediatric cancer is one of the most burdensome chronic diseases, necessitating a variety of severe medical interventions. As a result, the disease and its treatment cause numerous acute and long-term medical, psychological, and socioeconomic strains for young patients and their families. Therefore, psychosocial care using evidence-based interventions (EBIs) before, during, and after medical treatments is essential to ensure that patients receive adequate information and to minimize the adverse emotional and psychosocial impacts such as insecurity, fear, and shame. The present study reports the first promising results of applying cancer-specific psychosocial methods developed in the quality improvement project “My Logbook.” The four assessed tools are specifically designed to adequately prepare pediatric cancer patients for surgery, chemotherapy, radiotherapy, and stem cell transplantation. Self and proxy ratings were used to assess the patients’ subjective knowledge and emotional well-being before and after each intervention session. The results showed that patient-centered interventions using various creative and developmentally adapted methodologies (e.g., psychoeducation, crafting, games) have the potential to effectively enhance patient health literacy (*V* = 120.5, *p* < .001, *r* = 0.33) and well-being as manifested in more positive (slope = 0.121, *p* = .016) and less negative (slope =  − 0.350, *p* < .001) or neutral emotions (slope =  − 0.202, *p* = .002). These findings highlight the importance of developing and implementing psychosocial tools in pediatric oncology to prevent psychological overload and negative emotions and to increase subjective control beliefs, autonomy, and empowerment. Moreover, the effective application and systematic evaluation of evidence-based psychosocial tools can facilitate the establishment of standardized guidelines for psychosocial care in pediatric oncology. Thereby, the final goal is to ensure the quality of care and to use education to increase the quality of life for all pediatric cancer patients.

Trial registration: ClinicalTrials.gov Identifier: NCT04474678 (July 17, 2020)

## Background

Chronic illness confronts pediatric patients and their families with a myriad of medical as well as psychosocial burdens. The diagnosis and related socio-economic strains force affected children, adolescents, or young adults (CAYAs) as well as their families to grapple with rapid, severe changes to their lives, instability, and situations of crisis within the social system [1–5]. Moreover, pediatric cancer does not only have an acute medical, cognitive, and psychosocial impact. Still, it can bring about various long-term effects, including bodily deformations and impairment, reduced memory and attention function, deficient social integration, and unemployment [4, 6–9]. Overall, these manifold stressors related to the diagnosis and prognosis can lead to fear, anxiety, and guilt among the already burdened CAYAs [10, 11]. To facilitate successful coping and to increase compliance, international guidelines push for psychosocial care to become an integral part of pediatric oncological care, aiming to provide psychological guidance throughout all steps of the treatment process, starting at diagnosis and lasting until after-care [1, 12].

In addition to the aforementioned psychosocial stressors related to severe chronic illnesses, oncological disorders specifically require an array of invasive, painful, and potentially frightening medical interventions, necessitating appropriate psychosocial support and psychoeducation to prevent traumatization [3, 13, 14]. Neuro-oncological treatments include chemotherapy, radiotherapy, surgery, and stem-cell transplantation [1]. The need to undergo such treatments can have various consequences, such as physical side effects, pain, insomnia, and restrictions in daily life due to medical appointments and fatigue [15–18]. Due to the physical impact on the central nervous system, many patients also experience reduced cognitive performance in memory, speech, attention, and executive functioning [16, 19, 20]. Side effects such as hair loss or bodily deformation, as well as cognitive impairments and the inability to participate in school and social life, can further lead to impaired self-confidence and isolation as well as an emotional strain on patients and their families [10, 21, 22].

To counteract the treatment-related difficulties, tailored evidence-based interventions (EBI) have the potential to equip affected CAYAs and their families with powerful strategies to acquire the necessary resources for successfully coping with the oncological treatments [23, 24]. One critical element of EBIs is comprehensive psychoeducative preparation for complex oncological treatments. This includes the provision of all necessary information about the procedure and its mechanisms, the reasoning why it is necessary, and the steps the process entails. Importantly, this information needs to be presented in an engaging and age-appropriate manner and tailored to the patients’ and their family’s needs [25]. Moreover, patients must be provided with effective coping strategies to withstand the treatments. As summarized by Nunns et al. [26], these may include hypnosis and relaxation, or distraction and games. Before and after treatment, psychosocial support including counseling and emotional monitoring is essential to handling psychological distress as well as coping with physical effects after oncological interventions [27, 28]. The different types of interventions and their adequate use have been listed and described by guidelines published by authors such as Leiss et al. [29] or Nest et al. [27].

A steadily increasing number of EBIs has been developed and evaluated to work towards higher patient well-being and better patient education. However, the implementation of these interventions is slow and heterogeneous [30, 31]. Most psychosocial EBIs are specific to one issue and situation, such as reduced fear, cognitive training, improved manageability of administration, and increased compliance with medical interventions or family functioning [32]. Existing interventions often focus on increasing the patients’ health literacy, which has been associated with increased empowerment and autonomy, a better understanding of and control over the therapeutic process, and improved coping strategies facilitating a resilient outcome [33–35]. To fulfill the goals of patient education and improved patient well-being, interventions need to be constructed along with the patients’ actual needs [2, 24]. Ideally, patients and caregivers should not only be part of the evaluation of novel interventions as study participants but also following the concept of Patient and Public Involvement and Engagement (PPIE) be actively involved in the development of new interventions alongside researchers and healthcare professionals [e.g., 36–39]. Finally, for EBIs to be developed and effectively find their way into clinical practice, they should be composed considering the complex context of pediatric oncological care [30]. To facilitate the effective construction of patient-oriented, consensus-based, and feasible EBIs, the concept of quality improvement (QI) has been established. Applying methods such as the plan, do, study, and act cycles, the framework proposes for interventions to be developed with all relevant stakeholders and directly applied, evaluated, and improved within the predestined context [40, 41].

To date, no standardized EBIs have been developed that would adequately prepare pediatric cancer patients for the various burdening interventions to accompany them throughout the entire treatment process. To fill this gap, the QI project “My Logbook” project [42] aims to provide comprehensive standardized psychosocial guidance to pediatric patients by translating an evidence-based psychosocial guideline for pediatric oncological care [1] into a consensus-based, patient-centered training tool. The tool consists of a collection of topic booklets, each providing information on and interventions for a specific stage in the treatment process. The interventions included psychoeducation, reinforcement of psychosocial resources, and neuropsychological training and were evaluated in a multi-center study regarding the feasibility and efficacy of improving patients’ well-being and treatment-related knowledge. Since the first version of the “My Logbook” was developed in the context of pediatric neurooncology, four of the topic booklets specifically address invasive oncological interventions, namely radiotherapy, chemotherapy, stem cell transplantation, and neurooncological surgery. The present article aims to present and discuss the content and evaluation of these four topic booklets.

## Methods

### Study Procedure

The herein-described psychosocial tools to accompany pediatric oncological patients in four medical interventions are part of the “My Logbook” project, consisting of numerous equitable topic booklets, each constructed following the same procedure. A detailed description of the approach can be found in the corresponding protocol paper [43].

### Study Sample

**Fig. 1:**
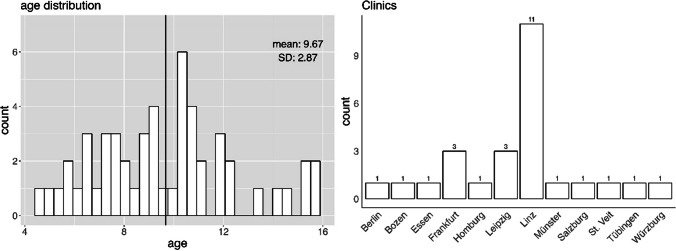
Age distribution of patients and participating clinics with participant count (except Vienna with *n* = 37)

The study sample consisted of *n* = 63 patients from *n* = 13 clinics across Germany, Italy (South Tyrol), and Austria. Specifically, the number of patients per topic booklet was “ABC of Chemotherapy”: *n* = 36, “My path through Radiotherapy”: *n* = 20, “Did you know? Everything about your operation”: *n* = 18, and “Mission stem cell transplantation”: *n* = 3. Patients’ mean age was *M* = 9.67 (SD = 2.87), with 52.9% being female (see Fig. 1). Sixty-five percent of patients stated their diagnosis. Of those, 41% have a brain tumor, 15% have leukemia, and 44% have a solid tumor. Fifty-one percent of patients have completed an operation, 46% have undergone radiation therapy, 78% chemotherapy, and 7% stem cell transplantation (Table 2).

Participants were given three main questionnaires: the Strengths and Difficulties Questionnaire (SDQ) [44, 45] on personal strengths and weaknesses, the KINDL-R [46] for assessing health-related quality of life, and the Health Literacy Scale (Gesundheitskompetenzskala) [47]. Most patients’ answers were in the normal range for all surveys. Regarding the SDQ, kids and their parents generally saw themselves as average. The KINDL-R showed similar results, with kids and parents rating themselves as average. Also, those who completed the Health Literacy Scale reported average health literacy. While about two-thirds of patients or their parents completed the SDQ (*n* = 42) or the KINDL (*n* = 40), only about one-third did so for the Health Literacy Scale (*n* = 22).

### Booklet Content

**Fig. 2:**
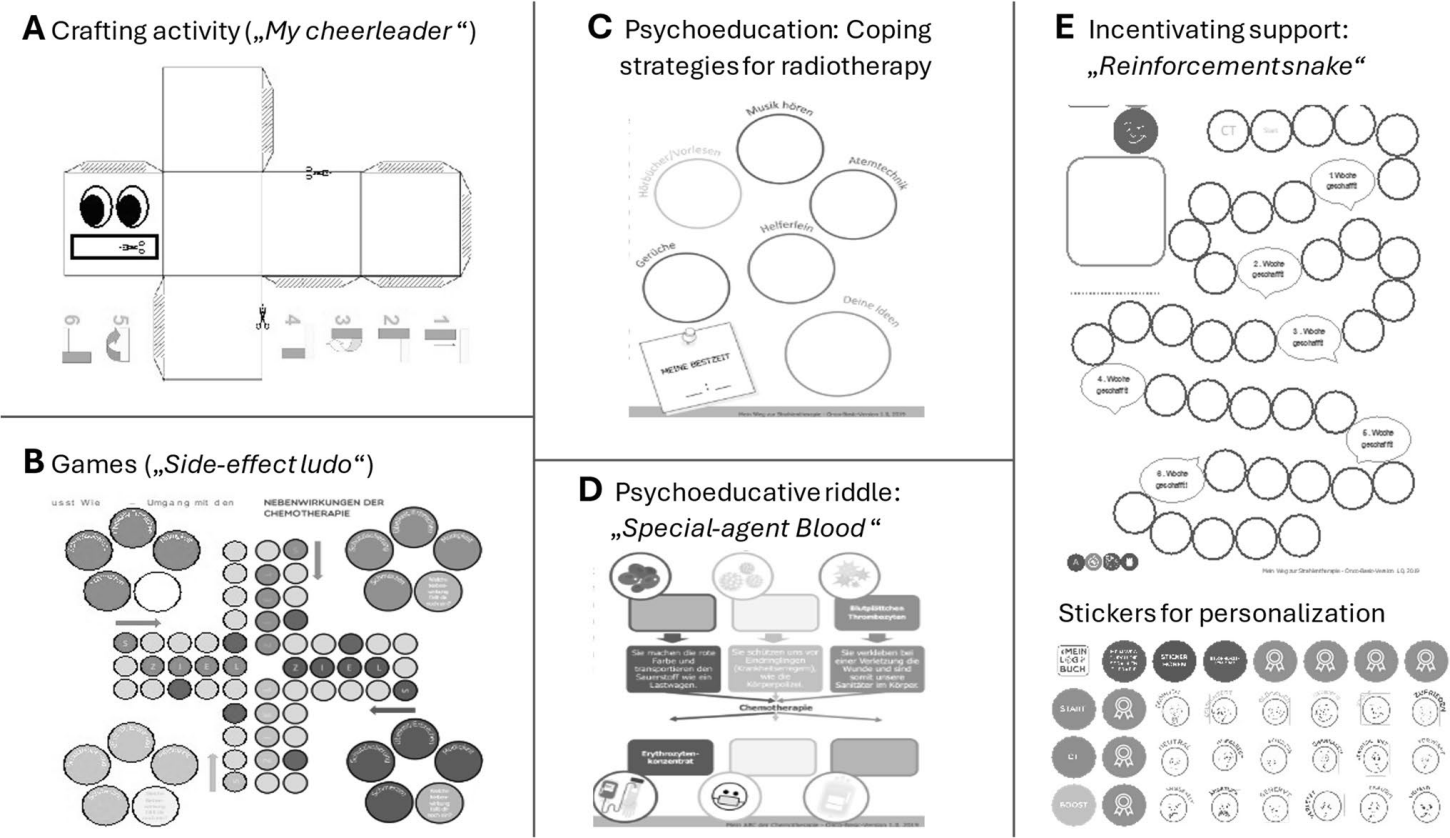
Exemplary methodologies used in the EBIs of the evaluated “My Logbook” topic booklets

Based on the collaboration of a local expert team and the evaluation by various international professionals, the content of each topic booklet was designed to provide patients and families with the necessary information to increase their health literacy regarding the intervention in question. Furthermore, EBIs to facilitate effective coping and strengthen personal resources were included. Each topic booklet starts with a general introduction, definition, and the basic information necessary for orientation and ends with a “now I know my way around” which is a checklist to conclude and reflect upon the learned content. Table 1 provides an overview of all methods included in each of the four topic booklets. Figure 2 shows examples of the different methodologies used in the EBIs of the different topic booklets.

**Table 1:** Psychosocial methods incorporated in each topic booklet

Topic booklet name	Methods
Did you know? – everything about your surgery [Weißt du schon – Alles über deine Operation]	• Game “Surgery Pro”: psychoeducation on experience with surgery, body parts, surgical instruments (collecting stickers) • Surgery passport: guidance through the surgical process • “Dream story”: Relaxation exercises to prepare for anesthesia • Post-surgery: information on waking up, potential complications, resources, and strategies for after-care
My ABC of Chemotherapy [Mein ABC der Chemotherapie]	• Psychoeduction: purpose of bodily cells, tumor development, purpose, modes of action, and duration of chemotherapy • Chemo process: discussion of the chemotherapy process and preparation for information and planning with medical staff • Chemotherapy plan: timeline with stickers to depict personal therapy plan • What to do?: discussion of necessary steps before, during and after chemotherapy • Special-agent blood: riddle about different blood cell types • “My cheerleader”: crafting a carton box depicting things that cause anger, worry, joy, pride, and positive memories • “Side-effect ludo”: a board game to discuss possible side effects and how to manage them
My way through radiotherapy [Mein Weg durch die Strahlentherapie]	• Psychoeduction: definition, equipment, process, duration of, and assessments before radiotherapy • Preparation: information on potential need for anesthesia and absence of parents during treatment; strategies for successful radiotherapy (relaxation, music, meditation, toys, …) • Radiotherapy agenda: personal timeline and list of appointments • Planning: discussion of necessary steps before, during, and after chemotherapy • Bonus task: visiting therapy location and getting to know appliances and team • Reinforcement snake: timeline to fill with stickers
Mission stem cell transplantation [Mission Stammzell-transplantation]	• “Ready for take-off” checklist: “space”-themed discussion of need for SCT, process, duration, medical team, coping strategies, resources, and emotions + decoration of personal SCT spaceship • Preparation: planet-themed discussion of necessary steps to be taken before, during, and after SCT (medication, hygiene, assessments) • Transplantation-planet: preparation for and description of isolation room • Planning daily structure during aplasia to reinforce empowerment and self-efficacy + additional crafting material and collecting cards on emotions, coping strategies, and practical information • “Escaping the labyrinth”: game to discuss regeneration and potential side/late-effects

### Data Analysis

Data analysis and creation of plots were conducted using the statistical software R (version 4.1.0) [48]. Data visualizations were generated using the ggplot2 package [49].

We used a generalized linear mixed model (GLMM) with Poisson regression to analyze the progression of positive, neutral, and negative emotions. The GLMM was implemented using the lme4 package (Bates et al., 2015). We also included the interaction terms between time points and neuropsychological predictors to investigate potential moderation effects.

A Wilcoxon signed-rank test was used to investigate the progression in self-reported knowledge. Additionally, a Wilcoxon rank sum test was employed to compare the differences between self-reported knowledge and the evaluations of knowledge by healthcare professionals.

## Results

### Intervention Characteristics

Most sessions were done by a psychologist (94%). For all four topic booklets, more than 70% of the goals that were suggested were reached (chemotherapy booklet = 71%, radiotherapy booklet = 75%, surgery booklet = 100%, stem cell booklet = 88%). On average, more than 60% of the sections in the booklets were completed (chemotherapy booklet = 67%, radiotherapy booklet = 62%, surgery booklet = 67%, stem cell booklet = 77%) (Table 2).

**Table 2:** Sample characteristics (*n* = 31)

Characteristics	n	%
Country		
Austria	12	39
Germany	16	52
Switzerland	1	3
Italy	2	6
Occupation		
(Clinical) psychologist	22	71
Psychotherapist	7	23
Medical doctor	2	6
Educator/pedagogue	1	3
Nurse	1	3
Art/music therapist	1	3
Survivor	1	3
Other	1	3
Focus of work		
Acute care	21	68
Out-patient aftercare	10	32
In-patient aftercare	6	19
Rehabilitation	4	13
Science	4	13
Other	1	3
Professional experience		
0 to 5 years	11	35
6 to 10 years	8	26
More than 10 years	12	39

### Subjective Expertise

**Fig. 3:**
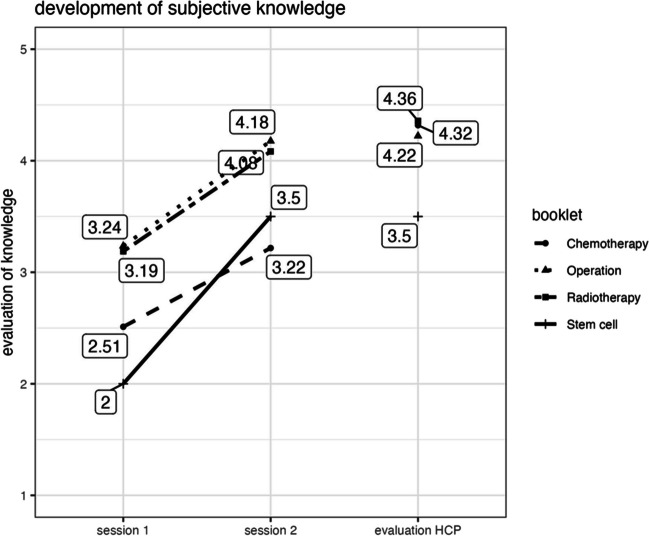
Development of subjective knowledge for patients (session 1 to session 2) and HCP’s knowledge evaluation at session 2

The study observed a statistically significant increase in self-reported knowledge between the first and second sessions (*V* = 120.5, *p* < 0.001, *r* = 0.33) across all four topic booklets combined. Also, there was a significant difference in self-reported knowledge between the second session and the knowledge evaluation provided by a healthcare professional (*W* = 1169.5, *p* = 0.030, *r* = 0.21). According to HCP evaluations, the topic booklets achieved many psychosocial goals determined during their development. The evaluations did not significantly differ between the topic booklets (*p* > 0.05) (Fig. 3).

### Emotional Well-being

**Fig. 4:**
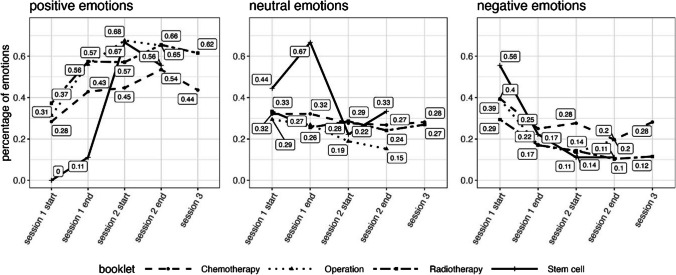
Development of positive, neutral, and negative emotions for topic booklets on chemotherapy, radiotherapy, and surgery. Due to the small sample size (*n* = 3), the booklet for stem cell therapy is not depicted

Positive emotions increased slightly throughout the sessions across the four topic booklets (surgery, CT, RT, SCT) (slope = 0.121, *p* = 0.016). Neutral emotions decreased significantly across the sessions (slope =  − 0.202, *p* = 0.002). Negative emotion development showed a statistically significant decrease across the sessions (slope =  − 0.350, *p* < 0.001). All three topic booklets by themselves showed a similar pattern concerning emotion development (see Fig. 4).

## Discussion

Our results describe the first evaluation of the cancer-treatment–specific topic booklets of the training tool “My Logbook,” which translates psychosocial care standards in pediatric oncology into a practical patient-centered tool. The tool aims to provide pediatric cancer patients with sufficient expertise in treatment-related information and coping strategies to facilitate successful compliance with severe medical treatments. This increase in self-efficacy and empowerment through knowledge, paired with continuous psychosocial mentoring and support, further aims to prevent negative emotions and decrease psychological distress during times of severe physical and mental strain. The HCP’s evaluation of achieved psychosocial goals using the “My Logbook” indicates the tool’s efficacy. However, the low number of completed sessions within the overall multi-center pilot study of the whole “My Logbook” project shows that there are still various barriers to overcome, hindering the implementation of adequate psychosocial care in pediatric oncology. Further evaluation, ideally in the form of randomized therapy optimization studies, is necessary to replicate this first evidence for the efficacy of the tool in a larger sample with the goal of facilitating the implementation of standardized high-quality psychosocial care for all patients in pediatric oncology.

One major aim of the “My Logbook” is to increase patients’ health literacy, which was evaluated using self and proxy ratings of the CAYAs’ subjective expertise. Although the small sample size does not allow for the detection of significant small effect sizes, the comparison of health literacy ratings before and after the interventions shows a clear tendency of increased knowledge, especially for the topic booklets on radiotherapy and surgery. This indicates that the content and methods of the topic booklets effectively facilitate the establishment of relevant basic knowledge and adequate coping strategies. Moreover, the HCPs’ ratings of the patients’ knowledge were in accordance with this, further supporting the tool’s efficacy in communicating necessary information in a patient-friendly and understandable manner. This illustrates that it is indeed possible to transmit even complex and sensitive information to children when using adequate methods. Thereby, it proved especially useful to use playful and creative methods and provide the affected CAYAs with sufficient time and space to get acquainted with the appliances and the medical staff that will carry out the treatment.

The second important goal of psychosocial care during pediatric oncological treatment is the prevention of emotional disturbances, which was evaluated using emotion ratings at the beginning and end of each session with the psychosocial professional. Despite the increased burden via medical treatments, the tendency of positive emotions to increase highlights the potential of psychosocial support and enhanced self-efficacy in reinforcing emotions such as pride and contentment under challenging situations. The significant decrease indicates that the Logbook can effectively reduce feelings of fear and anxiety via the thorough preparation and reinforcement of personal and social resources and training of effective coping strategies. Finally, the decreased tendency in neutral emotions might be explained by the patients’ increased ability to name and communicate emotions effectively.

The overall promising results of this first multi-center pilot study emphasize the potential of the My Logbook tool to facilitate the systematic implementation of psychosocial EBIs to improve patient education and well-being during active childhood cancer treatment. The study lays an important basis for the continuous improvement and implementation of the My Logbook. The hope is for the tool to be evaluated in an international treatment optimization study to systematically assess its benefits and shortcomings allowing for further improvement of the tool. As in all cancer care interventions the final aim is to create an effective and easily administrable tool with the potential to facilitate high-quality psychosocial care for all childhood cancer patients.

## Conclusion

The presented findings provide the first evidence that the evaluated pediatric cancer–specific psychosocial tools may effectively strengthen psychosocial well-being and subjective disease-related knowledge among pediatric oncological patients. The perspective of effective, comprehensive, and standardized support for this vulnerable demographic highlights the importance of continued development and implementation of the existing EBIs, such as the “My Logbook” tool. Thereby, it is critical that the applied patient-centered methodologies and psychoeducative information can be adapted to each patient’s developmental stage and individual needs, to ensure that the burdened CAYAs benefit to the highest possible degree and are not overwhelmed by the content. Thus, continuous evaluation, adaptation, and development of psychosocial tools in pediatric oncology have the potential to reinforce evidence-based standards of care in pediatric oncology to improve the quality of care. Such standardized high-quality psychosocial care would ensure that all pediatric cancer patients receive the information, support, and strategies they need to effectively cope with their disease, ultimately contributing to a higher quality of life.

## Data Availability

The datasets generated and/or analyzed during the current study are available from the corresponding author upon reasonable request.
